# Deregulated hedgehog pathway signaling is inhibited by the smoothened antagonist LDE225 (Sonidegib) in chronic phase chronic myeloid leukaemia

**DOI:** 10.1038/srep25476

**Published:** 2016-05-09

**Authors:** David A. Irvine, Bin Zhang, Ross Kinstrie, Anuradha Tarafdar, Heather Morrison, Victoria L. Campbell, Hothri A. Moka, Yinwei Ho, Colin Nixon, Paul W. Manley, Helen Wheadon, John R. Goodlad, Tessa L. Holyoake, Ravi Bhatia, Mhairi Copland

**Affiliations:** 1 Paul O’Gorman Leukaemia Research Centre, Institute of Cancer Sciences, College of Medical, Veterinary and Life Sciences, University of Glasgow, Glasgow G12 0XB, UK; 2 Division of Hematopoietic Stem Cell and Leukemia Research, City of Hope National Medical Centre, Duarte, CA, USA; 3 Novartis Institutes for Biomedical Research, Basel, Switzerland; 4 Histology, The Beatson Institute for Cancer Research, Garscube Estate, Switchback Rd, Glasgow G61 1BD, UK; 5 Department of Histology, Western General Hospital, Crewe Road South, Edinburgh EH4 2XU, UK; 6 Division of Hematology-Oncology, University of Alabama Birmingham, Birmingham, AL, USA

## Abstract

Targeting the Hedgehog (Hh) pathway represents a potential leukaemia stem cell (LSC)-directed therapy which may compliment tyrosine kinase inhibitors (TKIs) to eradicate LSC in chronic phase (CP) chronic myeloid leukaemia (CML). We set out to elucidate the role of Hh signaling in CP-CML and determine if inhibition of Hh signaling, through inhibition of smoothened (SMO), was an effective strategy to target CP-CML LSC. Assessment of Hh pathway gene and protein expression demonstrated that the Hh pathway is activated in CD34^+^ CP-CML stem/progenitor cells. LDE225 (Sonidegib), a small molecule, clinically investigated SMO inhibitor, used alone and in combination with nilotinib, inhibited the Hh pathway in CD34^+^ CP-CML cells, reducing the number and self-renewal capacity of CML LSC *in vitro*. The combination had no effect on normal haemopoietic stem cells. When combined, LDE225 + nilotinib reduced CD34^+^ CP-CML cell engraftment in NSG mice and, upon administration to EGFP^+^ /SCLtTA/TRE-BCR-ABL mice, the combination enhanced survival with reduced leukaemia development in secondary transplant recipients. In conclusion, the Hh pathway is deregulated in CML stem and progenitor cells. We identify Hh pathway inhibition, in combination with nilotinib, as a potentially effective therapeutic strategy to improve responses in CP-CML by targeting both stem and progenitor cells.

Chronic myeloid leukaemia (CML) is a clonal myeloproliferative disorder characterised by massive myeloid expansion, accumulation of differentiating granulocytic precursors and terminally differentiated effector cells leading to the key clinical features at presentation of marked peripheral blood granulocytosis, basophilia, splenomegaly and often thrombocytosis and anaemia^1^. Untreated, the clinical course of CML is one of inevitable progression from a stable chronic phase (CP) lasting about 5 years from diagnosis, where there is gradual accumulation of leukaemic myeloid progenitors, to accelerated phase characterised by accumulation and clonal evolution of increasingly primitive myeloid precursors in the blood or bone marrow (BM) before terminating in a blast crisis (BC) with rapid accumulation of immature myeloid or lymphoid precursors resembling acute leukaemia.

In optimally responding CP-CML patients, the majority have molecular evidence of persisting disease after prolonged tyrosine kinase inhibitor (TKI) therapy^2^,^3^. Furthermore, of those patients who achieve sustained molecularly undetectable leukaemia and discontinue TKI treatment the majority suffer molecular relapse^4^,^5^,^6^,^7^. One reason for disease persistence despite prolonged TKI therapy is that, while harbouring the BCR-ABL fusion protein, leukaemic stem cells (LSC) are resistant to pharmacologically achievable concentrations of TKI^8^,^9^,^10^. Evidence is now emerging to suggest that CML LSC may not be dependent on BCR-ABL kinase signaling for survival^11^,^12^,^13^. Therefore, in order to eradicate the leukaemic clone and cure CML, it is likely that alternative strategies specifically targeting the CML LSC population alone or in combination with conventional TKI will be required, thus achieving synthetic lethality through the abrogation of other critical survival pathways^14^.

One of the major pathways influencing stem cell self-renewal is the hedgehog (Hh) signaling pathway^15^,^16^,^17^. In embryogenesis, this pathway is critically involved in patterning but remains active in adult tissue where it contributes to tissue homeostasis, regeneration and healing^18^. There is accumulating evidence that Hh signaling plays a critical role in the pathogenesis of various haemopoietic malignancies^19^,^20^,^21^,^22^,^23^. Particular interest has focused on the role of Hh signaling in CML. Studies have shown that Hh signaling is increased in BCR-ABL^+^ progenitor cells and Hh signaling is further upregulated with disease progression^24^,^25^,^26^,^27^. Dierks *et al*. demonstrated that Hh signaling is upregulated in a pMSCV/Bcr-Abl/IRES-GFP retroviral vector murine model of CML and in their patient cohort, which was predominantly comprised of BC-CML, through upregulation of Smoothened (SMO)^27^. Experiments using murine models of CML have demonstrated that *Smo* deletion or pharmacological inhibition results in reduced LSC and a greatly reduced capacity to recrudesce the disease in secondary hosts^24^,^27^. The combination of standard TKI therapy with the naturally occurring SMO inhibitor cyclopamine resulted in the largest reduction in LSC *in vitro* and *in vivo* in murine experiments^24^,^27^. Therefore Hh signaling is active in murine models of CML and is critical to the maintenance and expansion of the disease clone in BC-CML.

LDE225 (Sonidegib; Novartis Pharma) is a synthetic, highly potent and selective, small molecule clinical SMO inhibitor resulting from the optimisation of a hit arising from a high throughput screening phenotypic assay designed to identify SMO inhibitors. LDE225 interacts directly with SMO, in a similar fashion to cyclopamine, to reduce expression of downstream Hh signaling targets^28^. LDE225 is effective in various Hh-dependent tumour models and inhibits downstream expression of Hh targets in cell lines, *in vivo* animal models and in patients, and is currently under clinical trial investigation both as a single agent^29^ and in combination (reviewed in^30^).

It is not clear to what extent Hh signaling is relevant in CP-CML, where stem cell-directed therapy might arguably be more effective in achieving disease eradication. Therefore, we set out to further define the role of Hh signaling in CP-CML and determine if inhibition of Hh signaling, most easily achieved through inhibition of SMO, using the SMO inhibitor LDE225 was an effective strategy to target CP-CML LSC. Using a combination of *in vitro* stem cell assays and *in vivo* murine models, this is the first study to robustly report efficacy for a SMO inhibitor, as a single agent, or in combination with a TKI, in models of CP-CML.

## Results

### The Hh pathway is expressed and active in LSCs in an *in vivo* murine model of CP-CML and in primary patient-derived CP-CML LSCs

**Figure 1:**
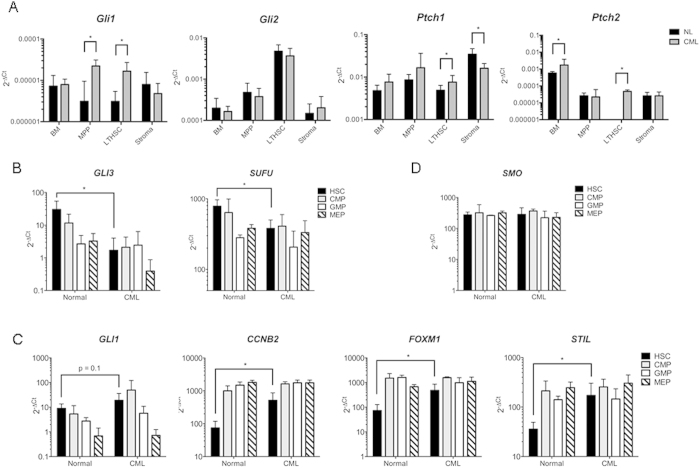
The Hh pathway is expressed and active in LSCs in an *in vivo* murine model of CP-CML and in primary patient-derived CP-CML LSCs. (**A**) Expression of Hh pathway genes *Gli1*, *Gli2*, *Ptch1* and *Ptch2* in total BM cells, BM MPP and LTHSC subpopulations, and in BM stromal cells from wild type (non-leukaemic; NL) and *Bcr-Abl*-expressing mice (n = 6–8 mice per arm). (**B–D**) Gene expression in sorted subpopulations (HSC, CMP, GMP and MEP) from human CD34^+^ haemopoietic cells from CP-CML patients (n = 6) at diagnosis compared with normal subpopulations (n = 3). Relative gene expression of (B) Hh pathway inhibitory molecules *GLI3* and *SUFU;* (**C**) downstream targets *GLI1, CCNB2, FOXM1* and *STIL;* and (**D**) positive Hh pathway regulator *SMO*. Results represent the mean ± SEM for multiple samples normalised to *B2m* (murine) and *GAPDH* and *HRPT1* (human); and are expressed as 2^−ΔCt^. Significance values; *p < 0.05.

Quantitative RT-PCR was performed to assess the relative gene expression of components of the Hh signaling pathway in the bone marrow (BM) of the CP-CML murine model *Scl-tTa-BCR-ABL*^31^. BM long-term haemopoietic stem cells (LTHSC) showed increased expression of Hh target genes *Gli1, Ptch1 and Ptch2* (all p < 0.05), compared to control (Fig. 1A). *Gli2* expression was unchanged. Similarly, *Scl-tTa-BCR-ABL* BM multipotent progenitors (MPP) showed increased expression of the Hh target gene *Gli1* (p < 0.05). In contrast stromal cells isolated from murine CP-CML BM demonstrated reduced expression of *Ptch1* with no change in *Gli1* expression. Additionally other Hh targets such as *Cdc2 and CcnB1* were also increased in CML LTHSC and MPP (data not shown). No difference in expression of *Ihh* expression was seen between CML and normal BM cells. *Dhh* and *Shh* were not detected (data not shown).

To better understand alterations in Hh signaling in primary human CML stem and progenitor cell populations, we sorted primary CP-CML or normal CD34^+^ cells into pure haemopoietic stem cell (HSC/LSC; lin^−^CD34^+^CD38^−^CD90^+^), common myeloid progenitor (CMP; lin^−^CD34^+^CD38^+^CD123^+^CD45RA^−^), granulocyte-macrophage progenitor (GMP; lin^−^CD34^+^CD38^+^CD123^+^CD45RA^+^) and megakaryocyte-erythroid progenitor (MEP; lin^−^CD34^+^CD38^+^CD123^−^CD45RA^−^) subpopulations by FACS. Each subpopulation was analysed by dual-fusion FISH (D-FISH) for the presence of the Philadelphia (Ph) chromosome and scored. Virtually all cells of all subpopulations in CP-CML were Ph^+^ by D-FISH analysis (Supplementary Table 1). There was no significant difference between subpopulations from individual patients. Greater than 87% of HSCs in all patients were Ph^+^, confirming that the translocation arises in the most primitive haemopoietic cells that can be isolated by surface phenotype, and is the dominant clone in all myeloid progenitor subpopulations. Furthermore, we assessed *BCR-ABL* gene expression in the different stem and progenitor cell populations and confirmed increased expression of *BCR-ABL* mRNA in the CP-CML LSC population relative to the other subpopulations (Supplementary Figure 1).

Gene expression of Hh pathway mediators and downstream targets was assessed in HSC, CMP, GMP and MEP subpopulations from CP-CML compared with normal samples (Fig. 1B–D). There was altered expression of several key regulators and targets of Hh signaling in CML LSC versus normal HSC. Most prominently we noted reduction in expression of the Hh pathway inhibitors *GLI3* and *SUFU* (both p < 0.05; Fig. 1B) in CP-CML LSC compared to normal HSC. We also observed up-regulation of downstream targets *CCNB2, STIL and FOXM1* (all p < 0.05; Fig. 1C) in CP-CML LSC. There was a trend for up-regulation of *GLI1* in CP-CML LSC, but this did not reach statistical significance (p = 0.1). In addition, higher levels of *GLI1* were maintained in primitive CML progenitors (CMP and GMP) compared to normal. There was no alteration in expression of *SMO* either between CP-CML and normal tissue or between haemopoietic progenitor subpopulations (Fig. 1D).

Thus, at the mRNA level, critical downstream targets and mediators involved in Hh signaling are deregulated in CP-CML both in the *Scl-tTa-BCR-ABL* murine model of CP-CML and in primitive CP-CML HSC derived from primary patient samples.

**Figure 2:**
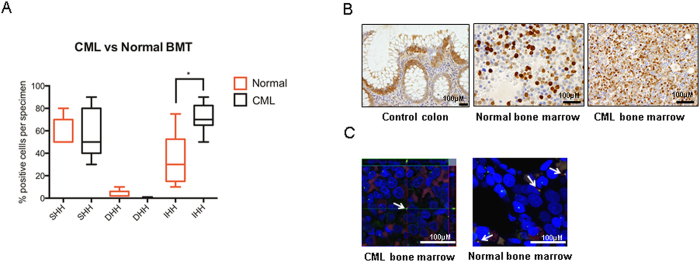
The Hh pathway is expressed at the protein level in human haemopoietic cells with the machinery (i.e. primary cilia) to enable canonical Hh signaling. (**A**) Percentage of SHH, DHH and IHH positive cells per bone marrow trephine (BMT) specimen in CP-CML (n = 7) compared to normal (n = 5). Results represent the mean ± SEM for multiple samples. Significance values; *p < 0.05. (**B**) Examples of BM trephine staining of SHH by immunohistochemistry. (**C**) Example of confocal images of primary cilia from BM trephine samples comparing CP-CML (n = 10) to normal controls (n = 10). There was no significant difference in the number of primary cilia observed between normal and CP-CML BM haemopoietic cells. Scale bar 100 μM in all images. Arrows highlight primary cilia demonstrated by co-localisation of α- and γ-tubulin.

To demonstrate Hh pathway protein expression and activity, immunohistochemistry (IHC) for ligands SHH, IHH and DHH was performed on BM trephines from CML patients and healthy individuals. There was no significant difference in expression of SHH or DHH between CML and healthy subjects (Fig. 2A). Interestingly, SHH showed both nuclear and cytoplasmic staining within trephine samples (Fig. 2B). A nuclear expression pattern has been previously described in subsets of cells in the CNS^32^. IHH was modestly increased in CP-CML (p < 0.05; Fig. 2A); DHH was not expressed.

In order to determine if SMO is a potential therapeutic target it was vital to determine if haemopoietic cells had the machinery, i.e. primary cilia, to enable canonical Hh signaling. Unmanipulated confocal images of BM trephines from both normal controls and CP-CML clearly show cilia to be present (Fig. 2C). Presence of primary cilia was at a low frequency in both the normal (n = 10) and CML (n = 10) samples. This is the first description of the presence of cilia within haemopoietic tissue and provides evidence that these cells are capable of canonical Hh signaling which may be targeted by SMO inhibition.

Thus, quantitative RT-PCR of CP-CML LSC from both a murine model and primary patient samples, and immunohistochemistry/immunofluorescence of primary human BM samples provide robust evidence for Hh pathway de-regulation in CP-CML and a rationale for exploring the efficacy of the clinically relevant SMO antagonist LDE225 as a Hh pathway inhibitor in CP-CML.

### The small molecule SMO antagonist LDE225 inhibits downstream Hh signaling *in vivo* in a CP-CML murine model and in primary CD34^+^ CP-CML cells

**Figure 3:**
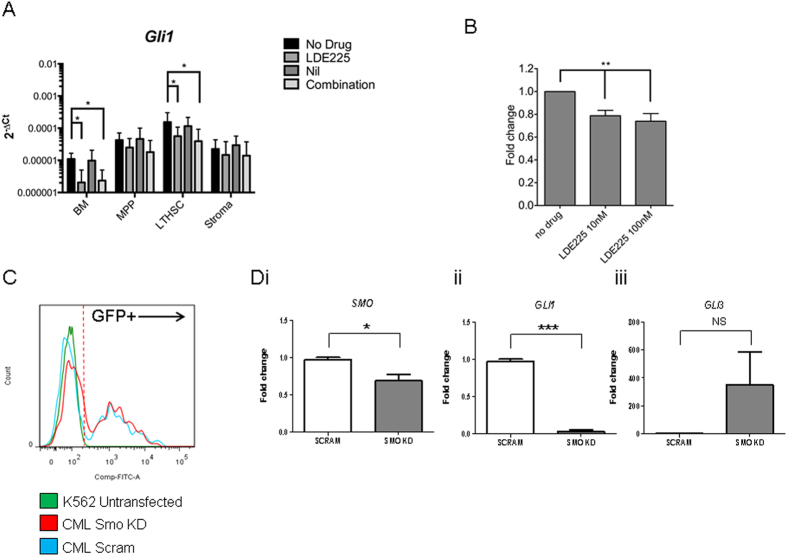
Pharmacologic and lentiviral SMO inhibition inhibits downstream Hh signaling in CP-CML cells. (**A**) Expression of *Gli1* in total BM cells, BM MPP and LTHSC subpopulations, and in BM stromal cells from *Bcr-Abl*-expressing mice (n = 8 per arm) treated with LDE225, nilotinib, LDE225 + nilotinib or vehicle alone for 5 days. Results represent the mean ± SEM for multiple samples. (**B**) Expression of *GLI1* following 72h exposure to LDE225 in CD34^+^ CP-CML cells (n = 7). Results represent the mean ± SEM for multiple samples. (**C,D**) Expression of targets and mediators of Hh signaling in CD34^+^ CP-CML cells following shRNA mediated SMO KD; (**C**) Representative flow cytometry histogram demonstrating comparative transfection efficiency of lentiviral vector containing GFP-SMO KD shRNA or GFP-Scram shRNA compared to untransfected cells. (**D**) Expression of *SMO* (Di), *GLI1* (Dii) and the Hh pathway inhibitor *GLI3* (Diii) in CD34^+^ CP-CML cells following shRNA mediated SMO KD normalised to GAPDH and compared to the scrambled control. Results shown represent the mean ± SEM for 3 independent transfections of different CD34^+^ CP-CML samples. Significance values; *p < 0.05; **p < 0.01; ***p < 0.005.

Studies have demonstrated that LDE225 has a low nM IC50 in cell lines^28^. Based on previous studies indicating that *GLI1* is an appropriate biomarker for Hh pathway activity^33^,^34^ and our own data showing up-regulation of *GLI1* in both primary human and murine CP-CML LSC, we sought to assess the effect of SMO inhibition with LDE225 on *GLI1* expression in CP-CML cells. *In vivo* treatment of *Scl-tTa-BCR-ABL* mice with LDE225 ± nilotinib resulted in inhibition of *Gli1* in CP-CML BM LTHSC (Fig. 3A). Treatment with nilotinib alone did not affect Hh-related genes, except cell cycle-related genes which may be the result of BCR-ABL inhibition (data not shown). The combination of LDE225 + nilotinib did not significantly increase inhibition of *Gli-1* (Fig. 3A) or other Hh-related genes compared to LDE225 alone (data not shown).

Primary CD34^+^ CP-CML cells were cultured in serum free media (SFM) ± LDE225 for 6, 24 and 72 hours (h). No alterations in *GLI1* were seen at 6 and 24 h (data not shown). At 72 h, while there was variability between the biological samples, *GLI1* was significantly downregulated following exposure to LDE225 (10 nM; 0.78-fold and 100 nM; 0.73-fold, respectively (p < 0.01; Fig. 3B).

To provide independent confirmation of the effect of SMO inhibition in CP-CML, lentiviral-mediated shRNA knockdown (KD) of *SMO* in primary CD34^+^ CP-CML cells was performed. Three independent primary CD34^+^ CML samples were transduced with a lentiviral vector expressing GFP and either *SMO* targeted shRNA or a scrambled control. Selection was based on GFP expression (Fig. 3C). The reduction in expression of *SMO* was modest in primary samples (mean 68% of control; p < 0.05; Fig. 3Di). Nevertheless, clear reduction in expression of *GLI1* was seen in all three samples (mean 3% of control; p < 0.005; Fig. 3Dii). Additionally, a trend towards increased expression of *GLI3* was seen (Fig. 3Diii).

Thus, pharmacologic (LDE225) or genetic inhibition (shRNA KD) of *SMO* leads to reduced expression of *GLI1*, a key target of Hh signaling, in primary CD34^+^ CP-CML cells and in a murine model of CP-CML.

### Inhibition of Hh signaling with LDE225 significantly reduces colony forming cell (CFC) re-plating efficiency of primitive human CP-CML cells

**Figure 4:**
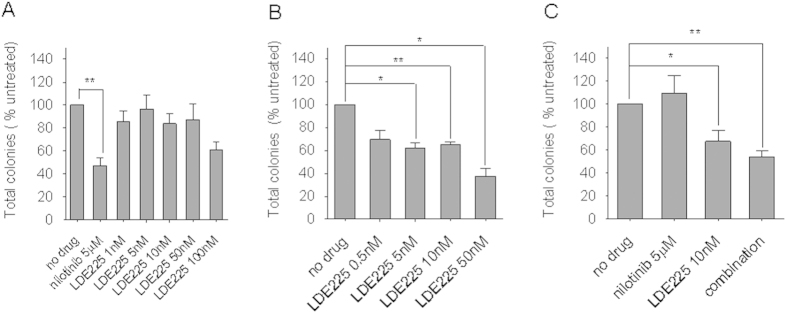
LDE225 alone or in combination with nilotinib reduces re-plating capacity of CD34^+^ CP-CML cells. (**A**) Relative CFC frequencies in each of the treatment arms normalized to an untreated control. Total number of secondary colonies formed at first re-plate from cells exposed to (**B**) escalating concentrations of LDE225 or (**C**) LDE225 10 nM, nilotinib 5 μM or both (combination), normalized to an untreated control. The results represent the mean ± SEM; n = 5. Significance values; *p < 0.05; **p < 0.01.

LDE225 does not induce pro-apoptotic or anti-proliferative effects on primary CD34^+^ CP-CML cells in short term *in vitro* culture (Supplementary Figure 2). To examine the behaviour of CP-CML cells following exposure to LDE225 in greater depth, we explored whether LDE225 affected primary colony formation and re-plating efficiency. Primary CD34^+^ CP-CML cells were cultured in SFM alone or in the presence of incremental concentrations of LDE225 ± nilotinib for 72 h. Following this, the cells were thoroughly washed to remove any remaining drug, inoculated into CFC assays and colonies assessed after 14 days (d). Increasing concentrations of LDE225 did not alter the size, number or type (GEMM, GM, CFU/BFU-E) of primary colonies formed (Fig. 4A).

To determine whether self-renewal behaviour had been altered by exposure to LDE225, we assessed secondary re-plating capacity of these colonies. We noted a significant reduction in re-plating capacity with increasing concentrations of LDE225 (p < 0.05) alone and in combination with nilotinib 5 μM (p < 0.01; Fig. 4B,C).

Thus LDE225, alone or in combination with nilotinib, did not affect primary colony formation but reduced secondary re-plating capacity, a measure of self-renewal activity in haemopoietic cells.

### LDE225 significantly reduces long-term culture-initiating cell (LTC-IC) frequency in CP-CML

**Figure 5:**
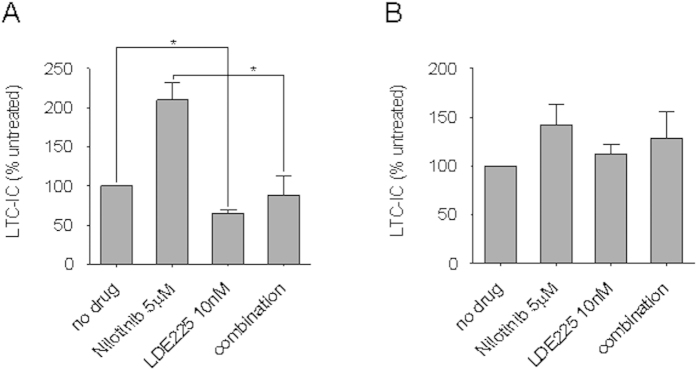
LDE225 alone or in combination with nilotinib significantly reduces LTC-IC numbers in primary CD34^+^ CP-CML samples. LTC-IC from (**A**) CD34^+^ CP-CML cells (n = 5) and (**B**) normal CD34^+^ haemopoietic cells (n = 3) were cultured in the presence of LDE225 10nM, nilotinib 5μM or both (combination). The results shown represent the mean ± SEM normalised to an untreated control. Significance values; *p < 0.05.

Primary CD34^+^ CP-CML cells or normal CD34^+^ haemopoietic cells were incubated in SFM with LDE225 10 nM ± nilotinib 5 μM for 72 h, washed and inoculated into a pre-prepared stromal co-culture. Compared with the untreated control, LDE225 resulted in lower LTC-IC recovery (p < 0.05; Fig. 5A). As previously shown with imatinib and dasatinib^35^, nilotinib demonstrated an enrichment of LTC-IC. Importantly, the addition of LDE225 to nilotinib led to a significant reduction in LTC-IC frequency relative to nilotinib alone, completely reversing the increase in LTC-IC seen with nilotinib (p < 0.05). Normal CD34^+^ haemopoietic cells demonstrated no alteration of LTC-IC abundance with LDE225, nilotinib or both relative to the untreated control (Fig. 5B).

Stem cell fate decisions are formulated in the context of complex micro-environmental conditions and signaling cascades^36^. In order to investigate the effect of long-term exposure to LDE225 ± nilotinib on CP-CML within a more complex microenvironment, we performed *in vitro* stromal co-culture in which CD34^+^ CP-CML cells were directly inoculated into supportive stromal co-cultures with incremental concentrations of LDE225 (10–100 nM) ± nilotinib 1 μM added to the stromal co-culture media. There was a significant reduction in LTC-IC numbers in the LDE225 arms (p < 0.05-p < 0.01) (Supplementary Figure 3A). Additionally, 5 weeks of continual exposure to nilotinib did not eradicate the LTC-IC population (28% of the untreated control remaining). Interestingly, in the experimental arms containing the combination of LDE225 + nilotinib, there was a trend to further reduction in the LTC-IC recovery suggesting that LDE225 targets a population of TKI-insensitive CP-CML cells or exerts additional anti-leukaemic effects by disruption of the microenvironment, although this was not evident microscopically with the stromal cell layers remaining intact throughout culture. (Supplementary Figure 3B).

Thus LDE225 alone or in combination with nilotinib reduced the primitive leukaemic population as measured by LTC-IC and reduced self-renewal activity as evidenced by re-plating capacity, suggesting that SMO inhibition targets the TKI-insensitive primitive stem/progenitor cell population in CP-CML.

### Effect of LDE225 in combination with nilotinib on human CML LSC capable of engrafting immunodeficient mice

**Figure 6:**
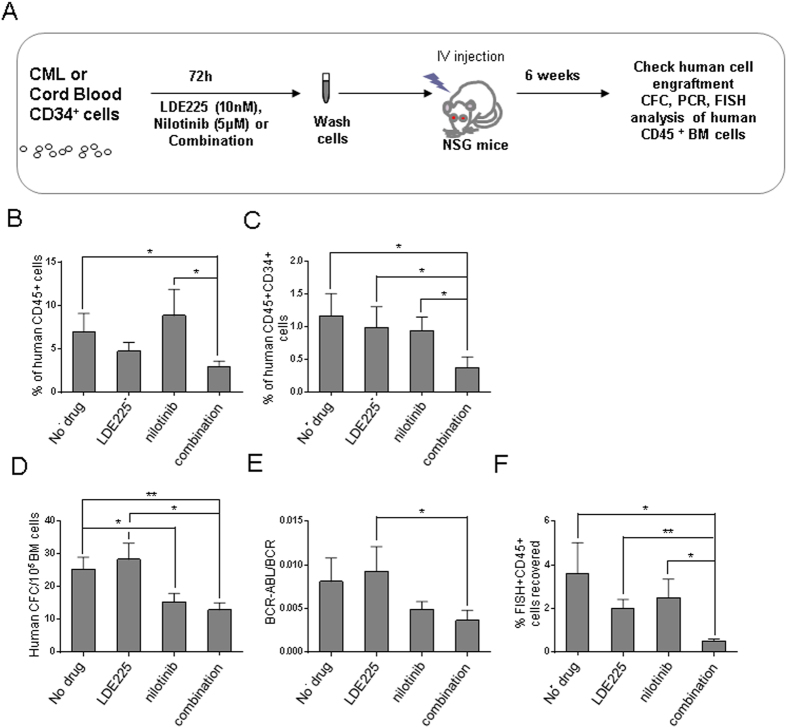
Combination of LDE225 + nilotinib reduces CML LSC capable of engrafting NSG mice. (**A**) NSG mice received CD34^+^ cells from CML patients (2 × 10^6^ cells per mouse) cultured for 72h in the presence of LDE225 (10nM), nilotinib (5μM), combination (10nM LDE225 + 5μM nilotinib) or vehicle alone (no drug control) by tail vein injection. Mice were euthanized after 6 weeks and marrow contents of femurs, spleen cells and blood cells were obtained. The percentage of human (**B**) CD45^+^ cells and (**C**) CD45^+^ CD34^+^ cells engrafted in the BM. (**D**) CFC frequencies within human CD45^+^ cells isolated from the BM of mice and grown in methylcellulose progenitor assays. (**E**) *BCR-ABL* mRNA levels in BM cells obtained from mice at 6 weeks post-transplantation (normalised to *BCR* mRNA levels). (**F**) The percentage of CD45^+^ BCR-ABL^+^ cells measured by FISH in BM cells obtained from mice at 6 weeks post-transplantation. Results shown represent the mean ± SEM for multiple samples. Significance values; *p < 0.05; **p < 0.01; n = 6–8 mice per treatment arm.

We tested the effect of LDE225 ± nilotinib on CD34^+^ CP-CML and normal cells capable of engraftment in NSG mice (Fig. 6A). We observed reduced engraftment of human CP-CML CD45^+^ cells treated with the combination of LDE225 + nilotinib compared with untreated or single treatment arms (Fig. 6B). Human CD45^+^ CD34^+^ cells and CFC were also reduced in the marrow of mice receiving cells treated with LDE225 + nilotinib compared with untreated, single agent LDE225 or nilotinib (Fig. 6C,D). qRT-PCR analysis confirmed that *BCR-ABL*^+^ LSC contributed to engraftment in all experimental arms, and *BCR-ABL* mRNA levels were reduced in CD45^+^ cells obtained from the BM of mice receiving CML cells treated with the combination of LDE225 + nilotinib, compared with untreated or single agent LDE225 (Fig. 6E). FISH analysis showed a significantly reduced proportion of *BCR-ABL*^+^ human cells engrafted in mice receiving cells treated with the combination of LDE225 + nilotinib compared to untreated controls and single agent arms (*p < 0.05; **p < 0.01; Fig. 6F). Cord blood CD34^+^ cells did not show changes in engraftment following treatment with nilotinib, LDE225 or LDE225 + nilotinib (Supplementary Figure 4). These results show that LDE225 + nilotinib selectively targets primitive CP-CML cells capable of *in vivo* engraftment.

### Effect of *in vivo* treatment with LDE225 in combination with nilotinib on LSC in a transgenic *BCR-ABL* mouse model of CML

**Figure 7:**
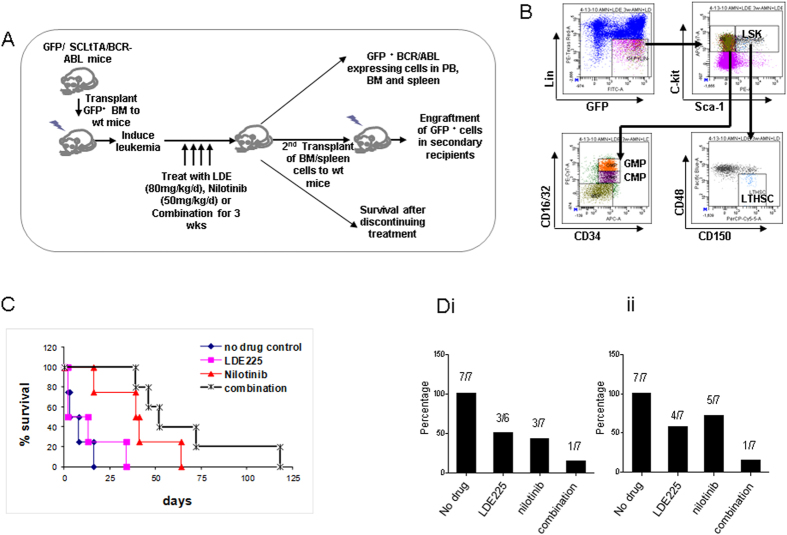
LDE225 + nilotinib administration significantly reduces leukaemia stem and progenitor cells in the spleen of BCR-ABL expressing mice. (**A**) Schema for experiments using the EGFP^+^ /*SCLtTA/TRE-BCR-ABL* mouse model to assess drug efficacy *in vivo*. (**B**) Representative FACS plot for LSK and LTHSC cells. (**C**) Survival of mice after discontinuation of treatment. (**D**) Results of transplantation of BM and spleen cells obtained after treatment (cells pooled from 8 mice, 5 × 10^6^ cells injected per recipient mouse, 8 mice per condition for both BM and spleen cells) into wild-type (wt) FVB/N mice. WCC and engraftment was monitored by drawing PB every 4 weeks. The fraction of recipient mice showing evidence of leukemia within 16 weeks after secondary transplantation was shown for (i) BM cells and (ii) spleen cells. Results represent the mean ± SEM for multiple samples. Significance values; *p < 0.05; **p < 0.01; n = 8.

Low levels of engraftment of CD34^+^ CP-CML cells in the NSG mouse model limit its utility for *in vivo* drug treatment studies. Therefore we utilised the transgenic *Scl-tTa-BCR-ABL* mouse model^31^ to investigate the effect of *in vivo* treatment with LDE225 ± nilotinib on CP-CML LSC (Fig. 7A,B).

There was no difference in the weight of the nilotinib or combination treated mice compared with the untreated cohort, however there was a small, non-significant incremental reduction in mean weight in the cohort treated with LDE225 alone over the 3 week treatment period (Supplementary Figure 5). Nilotinib alone resulted in a reduction of splenic GMP and CMP, but not LTHSC (Supplementary Figure 6Ai–iii). Single agent LDE225 reduced splenic LTHSC, but did not significantly reduce GMP and CMP. LDE225 + nilotinib significantly reduced GMP, CMP and LTHSC in the spleen. In contrast, in the BM of mice treated with LDE225, nilotinib or the combination, there were no significant changes in the numbers of GMP, CMP and LTHSC (Supplementary Figure 6Bi–iii).

A subset of mice were followed after completion of treatment, and survival off treatment was monitored for 120d (Fig. 7C). Control mice that survived the 3 week treatment period died within a further 16d. Improved survival after discontinuation of treatment was seen in mice treated with nilotinib (median survival 40d), and further trend towards increased survival was shown in mice treated with the combination of LDE225 + nilotinib (median survival 52d) (no treatment versus combination p < 0.01; nilotinib versus combination p = 0.15).

Bone marrow cells and spleen cells from a subset of treated mice were transplanted into secondary recipient mice. Transplantation of either BM or spleen cells from mice treated with LDE225 + nilotinib resulted in reduced WCC and reduced leukaemia development in secondary recipients compared to LDE225 or nilotinib alone (Fig. 7D). These results suggest that the combination of LDE225 + nilotinib reduces the number of BM and splenic LSC capable of engraftment in secondary recipients and causing disease relapse. Since the numbers of phenotypically identifiable BM LTHSC were not reduced after LDE225 treatment (Supplementary Figure 6Biii), these results suggest that LDE225 treatment results in reduced LTHSC self-renewal or engraftment potential.

## Discussion

There have been major advances in the treatment of CML in recent years with the development of imatinib^37^ and, more recently, dasatinib^38^, nilotinib^39^, bosutinib^40^ and ponatinib^41^. These newer compounds target the majority of imatinib-resistant mutations and, in the case of dasatinib, reach further into the LSC compartment^9^,^10^. However, despite increasingly potent inhibition of BCR-ABL, quiescent CML LSC remain insensitive to these compounds. Thus, there is mounting evidence that strategies to target both quiescent LSC and proliferating cells are required to eradicate CML^2^,^6^,^7^,^8^,^10^,^42^,^43^.

Chronic myeloid leukaemia LSC may not be dependent on BCR-ABL kinase signaling for survival^11^,^13^. Therefore, in the majority of patients, it is unlikely that the CML LSC can be completely eradicated through the use of BCR-ABL kinase inhibitors alone. This hypothesis is further supported by observations from our group and others that BCR-ABL-targeted therapies such as imatinib, dasatinib or nilotinib fail to eradicate CML LSC *in vitro*^8^,^9^,^10^,^42^.

Our data, along with those of Radich *et al*.^26^, Dierks *et al*.^27^, Zhao *et al*.^24^, Alonso-Dominguez *et al*.^44^ and Sadarangani *et al*.^45^ show that the Hh pathway is a relevant therapeutic target in CML. Radich *et al*. demonstrated that the effector of Hh signaling, *GLI2*, was upregulated, at least at the mRNA level, with disease progression from CP to BC^26^. Similar findings have also recently been reported in acute myeloid leukaemia by Wellbrock *et al*. with high levels of *GLI1* or *GLI2* identified as negative prognostic markers and direct inhibition of GLI1/2 exerting antileukaemic effects^34^. In our studies, increased *GLI1* expression was seen in CP-CML LSC and there was retention of higher levels of *GLI1* in primitive progenitor populations (CMP, GMP) compared to normal. *GLI1* is widely utilised as a reporter of Hh activity as it is regulated at the gene expression level and expression is responsive to Hh signaling^46^. We found expression of *GLI2* to be at the limits of detection in both normal HSC and CP-CML LSC cells (data not shown). Alonso-Dominguez *et al*. provided evidence that high *PTCH1* expression in mononuclear cells at diagnosis predicts imatinib failure in CP-CML and identifies a cohort of patients that may benefit from a second generation TKI as first-line therapy^44^. Our data demonstrated that *Ptch1* and *Ptch2* were significantly increased in murine LTHSC (Fig. 1A) and there was a trend for increased *PTCH1* in CML LSC (data not shown), however this did not predict imatinib failure. These discrepant results may be due to the different cell populations analysed (CP-CML LSC versus mononuclear cells).

The presented results indicate that the main negative regulators of Hh signalling, *GLI3* and *SUFU* are downregulated with degree of maturity in normal haemopoiesis, but stably expressed at a low level in CP-CML (Fig. 1B). We saw no difference in expression of SMO across subpopulations (Fig. 1D). These are intriguing results; the lack of difference in expression of the key positive regulator of Hh signalling *SMO* is in contrast with Dierks *et al*. who demonstrated that *Smo* expression was upregulated at the RNA and protein level in murine *BCR-ABL*^+^ cells^27^, providing a possible mechanism for Hh over activity in CML cells. Our results hint toward an alternative, cell autonomous mechanism of activation relating to reduced expression of key negative regulatory elements. When *SUFU* and *GLI3* are downregulated, the result would likely be to make a cell more sensitive to Hh signalling and the production of a stronger and more prolonged response^47^. This would require further work to confirm differential expression at the protein level but is an area of great mechanistic interest. Expression of several other targets of Hh signaling were differentially expressed between normal HSC and CP-CML LSC. These include *FOXM1*, *CCNB1/2* and *STIL*. Other signalling pathways converge on these molecules and expression may also be driven by other factors. For example, FOXM1 is a transcription factor with a role in cell proliferation and DNA repair, and in addition to Hh signalling, is regulated by input from various other pathways at a transcriptional level e.g. FOXO3A^48^, p53 and E2F1^49^. STIL is involved in haemopoiesis and is regulated by various factors including GATA transcription factors^50^.

Zhao *et al*. and Dierks *et al*. clearly demonstrated that Smo inhibition in murine models of CML, either by genetic knock-out or by pharmacological inhibition with the non-clinical SMO inhibitor cyclopamine, potently inhibited the propagation of leukaemic cells and the reconstitution of disease in secondary transplant models leading to prolonged survival of transplant recipients^24^,^27^. Both groups also provided evidence of a similar effect in primary BC-CML cells in *in vitro* surrogate assays of clonogenic potency. More recently, Sadarangani *et al*. demonstrated that BC-CML LSC could be targeted using the SMO antagonist PF-04449913, and this acted synergistically with BCR-ABL inhibition to reduce BC-CML LSC survival and self-renewal, likely through downregulation of *GLI2*^45^. Further recent studies have shown evidence for efficacy of dasatinib in combination with the SMO inhibitor GDC-0449 in *BCR-ABL*+ cell lines^51^ and ponatinib in combination with GDC-0449 in resistant *BCR-ABL*+ cells expressing the T315I mutation^52^. From a clinical perspective, treating BC-CML and T315I -mutated CML remain important, but the advent of TKI therapy has allowed increasing numbers of patients to remain in CP indefinitely with relatively low risk of progression albeit at the price of continuous treatment, due in part to LSC persistence. Therefore there is a clear imperative to attempt to find more effective ways of targeting the malignant LSC at an earlier stage (i.e. CP-CML) prior to progression to advanced phase.

Here we provide evidence that targeting the Hh pathway using the clinically investigated SMO inhibitor LDE225 in combination with nilotinib is an effective strategy for targeting CP-CML LSC. We show that LDE225 + nilotinib results in reduced CFC re-plating capacity and LTC-IC potential, reduced engraftment of human CD34^+^ CML cells in immunodeficient mice and prolonged survival with reduced leukaemogenic potential in the transgenic *Scl-tTa-BCR-ABL* mouse model that is more representative of CP-CML. It is noteworthy that LDE225 selectively inhibits self-renewal of LSC with minimal effects on apoptosis and proliferation of committed progenitors, differentiating this agent from most other anti-leukaemia agents currently being tested. Our observation of primary cilia in both normal haemopoietic and leukaemic cells indicates that the Hh pathway may be acting via canonical signaling, although we cannot exclude that activation of downstream Hh signaling results from cross-talk between the Hh pathway and other self-renewal pathways^25^. In contrast to Dierks *et al*. who indicated that Hh pathway activation in *BCR-ABL*+ leukaemogenesis was via upregulation of SMO^27^, we found no evidence for this; with no increased expression of SMO at the gene or protein level in CP-CML. A possible reason for this is the different *in vitro* cell models and murine models used in addition to the different stages of CML under analysis. The ability of the SMO inhibitor LDE225 in combination with nilotinib to selectively target quiescent LSC is an important step in the development of LSC-directed therapies.

This is the first study to demonstrate that a SMO inhibitor suitable for clinical development has efficacy against CP-CML LSC both *in vitro* and *in vivo*, and provided preclinical support for clinical trials of LDE225 (Sonidegib) in combination with nilotinib in CP-CML patients who have failed prior therapy with other BCR-ABL tyrosine-kinase inhibitors (NCT01456676). However, as with a similar study combining a SMO inhibitor with dasatinib^53^, this clinical trial has terminated early due to lack of efficacy and tolerability^54^. The heterogeneity of Hh pathway gene expression and response to the SMO inhibitor LDE225 indicate that further studies are required to identify if there are particular subpopulations of patients more likely to benefit from combination therapy with a SMO inhibitor ± TKI and if there are downstream Hh pathway targets amenable to therapy with less toxicity.

## Materials and Methods

### Patient samples

Patient samples were peripheral blood or leukapheresis products taken at time of diagnosis with CML, with written informed consent obtained from all subjects in accordance with the Declaration of Helsinki, and approval of the Greater Glasgow and Clyde National Health Service Trust Institutional Review Board. Samples were enriched for CD34^+^ cells using the CliniMACS (Miltenyi Biotec) immunomagnetic beads system. Samples enriched for CD34^+^ cells were cryopreserved in 10% DMSO (Sigma-Aldrich) and 5% human albumin solution (Baxter Healthcare) in liquid nitrogen until required.

### Primary cell culture

Cryopreserved primary CD34^+^ cells were recovered from liquid nitrogen as previously described^10^ and cultured overnight in a humidified incubator at 37 °C with 5% CO_2_ in SFM consisting of Iscove Modified Dulbecco Medium (Sigma-Aldrich) containing serum substitute (bovine serum albumin, insulin, transferrin [BIT]; Stem Cell Technologies), 0.1 μM 2-mercaptoethanol (Sigma-Aldrich), penicillin-streptomycin, L-glutamine plus a high concentration growth factor (5GF) cocktail containing 100 ng/mL Flt3-ligand (Flt3-L), 100 ng/mL stem cell factor (SCF), 20 ng/mL interleukin-3 (IL-3), 20 ng/mL IL-6 (Stem Cell Technologies) and 20 ng/mL granulocyte-colony stimulating factor (G-CSF; Chugai Pharma) to maximize cell recovery. In selected experiments following overnight culture, cells were washed and cultured in SFM supplemented with 5GF cocktail, a physiological growth factor cocktail comprising 5 ng/mL Flt3-L, 5 ng/mL SCF, 1 ng/mL IL-3, 1 ng/mL IL-6 and 1 ng/mL G-CSF (LGF) or SFM alone.

### Reagents

LDE225 and nilotinib were kindly provided by Novartis Pharma. Ten millimolar stock solutions were prepared in DMSO (Sigma-Aldrich) and stored at −20 °C. Dilutions of these stock solutions were freshly prepared for each experiment in appropriate cell culture media.

### Immunocytochemistry of paraffin-embedded, decalcified bone marrow trephines

Sections were mounted on glass slides, deparaffinised and rehydrated. Antigen retrieval was performed according to standard protocols. Sections were subsequently washed in PBS/Tween before serially adding antibodies and incubating for 1 h at room temperature (RT) or overnight at 4 °C. Supplementary Table 2 shows antibody details. Slides were mounted with DAPI (Invitrogen) and visualised with a confocal scanning microscope (Zeiss). Positive and negative controls were included with each experiment; settings were adjusted between samples.

### Immunocytochemistry of paraffin-embedded, decalcified bone marrow trephines

Single antibody detection was accomplished using a Leica Bond III immunostainer. All antibodies were optimised prior to use on control material as specified by product datasheet. Supplementary Table 3 shows antibodies details.

### Fluorescence activated cell sorting (FACS) strategy

Cells were either used as a bulk CD34^+^ enriched population or stained with lineage cocktail, anti-CD34, CD38, CD90, CD45RA and CD123 (all BD Biosciences) prior to sorting into HSC (lin^−^CD34^+^CD38^−^CD90^+^), CMP (lin^−^CD34^+^CD38^+^CD123^+^CD45RA^−^), GMP (lin^−^CD34^+^CD38^+^ CD123^+^CD45RA^+^) MEP (lin^−^CD34^+^CD38^+^CD123^−^CD45RA^−^) populations using a FACS Aria flow cytometer (BD Biosciences).

### Proliferation/apoptosis/cell cycle analysis

CD34^+^ CP-CML cells were seeded in SFM alone ± LDE225 ± nilotinib and cultured for 24–72 h prior to assessment. Proliferation was measured using colorimetric assessment of BrDU incorporation (Roche). Proportion of viable cells versus those in early and late apoptosis was assessed by flow cytometry using annexin V–FITC and 7-amino-actinomycin D (7-AAD, Via-Probe solution; both from BD Biosciences) according to the manufacturer’s instructions. Cell cycle status was assessed as previously described using Ki67 (FITC; BD Biosciences) expression and 7-AAD incorporation^55^.

### CFC assay/re-plating assay

CD34^+^ CP-CML cells were seeded in SFM ± LDE225 ± nilotinib and cultured for 72 h then washed three times, inoculated at a concentration of 4 × 10^3^/ml into methylcellulose supplemented with growth factors (Methocult H4034; Stem Cell Technologies) and cultured in duplicate for 14d prior to colony assessment. Following assessment, at least 20 colonies (granulocyte-erythroid-megakaryocyte-macrophage [GEMM] or granulocyte macrophage [GM]) colonies were plucked from each experimental arm and serially re-dispersed in Methocult with secondary and tertiary colony formation assessed after 7d intervals.

### LTC-IC assay

Primary CD34^+^ normal and CP-CML cells were cultured in SFM ± LDE225 ± nilotinib for 72 h. Following this, they were thoroughly washed and inoculated into pre-prepared long term cultures comprising a stromal feeder layer (a 1:1 mix of irradiated (80 Gy) SL/SL and M210B4 murine fibroblasts) in long term myeloid culture medium (MyeloCult supplemented with hydrocortisone; both Stem Cell Technologies) as previously described^35^. These cultures were maintained for 5 weeks with 50% media changes performed weekly. Following this, the contents of the wells were harvested and cells counted prior to seeding into Methocult to perform CFC assays as described above.

### Long term stromal co-culture

CD34^+^ CP-CML cells were inoculated directly into pre-prepared stromal co-cultures, as described above, in the presence of LDE225 ± nilotinib. Cultures were maintained for 5 weeks with 80% media changes and addition of fresh drug weekly. Co-cultures were examined weekly by microscopy to ensure that the stromal layer remained morphologically normal and adherent. After 5 weeks, CFC assays were performed as described.

### Assessment of gene expression by qRT-PCR

RNA extraction was performed using the Qiagen RNEasy Minikit according to the manufacturer’s instructions. Reverse transcription was performed using the high capacity cDNA synthesis kit (Applied Biosystems). Quantitative RT-PCR was performed using the Taqman system on a Taqman 7900 instrument (Applied Biosystems). Gene expression was determined relative to *GAPDH* and expressed either as 2^−ΔCt^ or compared to an untreated calibrator (2^−ΔΔCt^)^56^.

### Assessment of gene expression in human and murine haemopoietic stem and progenitor cell subpopulations using Fluidigm Biomark

RNA was extracted from human and murine subpopulations, reverse transcribed, and 14 cycles of gene-specific amplification performed using the Applied Biosystem pre-amplification kit with relevant Taqman probe sets (Supplementary Tables 4 and 5). Following amplification the resultant products were loaded in triplicate onto pre-primed 48 × 48 Fluidigm microfluidic dynamic arrays and analysed according to the manufacturer’s instructions. Gene expression was determined relative to *GAPDH, HRPT1* or *B2m*.

### Fluorescence *in situ* hybridisation (FISH)

Fluorescence *in situ* hybridization was performed with the LS1 BCR-ABL Dual Color FISH probe (Abbott Diagnostics) according to the manufacturer’s instructions.

### Lentiviral ShRNA construction, production and transfection of primary CD34^+^ CP-CML cells

A hairpin against human *SMO* mRNA in a PLKO.1 plasmid was obtained from Open Biosystems (TRCN0000014367); (CCGGCCTGACTGTGAGATCAAGAATCTCGAGATTCTTGATCTCACAGTCAGGTTTTT), and was excised using Nde and SpeI restriction enzyme digestion and ligated into a PLKO.1-GFP tagged plasmid. PLKO.1-scram-GFP plasmid containing a non-targeting hairpin sequence acted as a control. HEK-293 cells were transfected with PLKO.1-ΔSMO-GFP or PLKO.1-scram-GFP control plasmid in the presence of HIV-1, VSV-g accessory plasmids and CaCl_2_. Virus laden supernatant was collected (DMEM supplemented with 20% FCS)^57^,^58^. For primary cell transfection, CD34^+^ CP-CML cells were removed from cryogenic storage and cultured overnight in SFM + 5GF. Thereafter, primary CD34^+^ CP-CML cells were spinoculated in freshly collected viral medium supplemented with Transdux™ (Cambridge Bioscience) at 400 × g for 90 mins at 24 h, 48 h and 72 h for a total of three rounds of transduction. Following this, CD34^+^ cells were washed and re-suspended in SFM + 5GF for 48 h prior to sorting. GFP^+^ cells were FACS sorted for subsequent RNA isolation. qRT-PCR was performed to assess relative abundance of Hh related mRNA.

### Human CD34^+^ Cell Engraftment in Immunodeficient Mice

The NSG mouse model (Jackson Laboratory) was used to assay human HSC/LSCs with *in vivo* engraftment capacity (SCID-repopulating cells or SRC). CD34^+^ CP-CML cells (2 × 10^6^ cells/ mouse) or cord blood (CB) CD34^+^ cells (1 × 10^5^ cells/mouse) were cultured for 72 h without drug (control), or with LDE225 (10 nM) ± nilotinib (5 μM), at 37 °C in a humidified atmosphere with 5% CO_2_ in SFM + GFs as previously described^59^. Cells were harvested, washed and transplanted via tail vein injection into sublethally irradiated (300 cGy) 8 week old NSG mice. Mice were euthanised after 6 weeks and marrow contents of femurs were obtained. To assess human cell engraftment, cells were labeled with anti-human CD45, CD34 and CD33 antibody prior to analysis by flow cytometry. Human CD45^+^ cells were selected using immunomagnetic columns and analyzed in CFC assays and by FISH and qRT-PCR for the *BCR-ABL* gene rearrangement. All experimental procedures were carried out in accordance with federal guidelines and protocols approved by City of Hope’s Institutional Animal Care and Use Committee.

### Analysis of Transgenic EGFP^+^/*SCLtTA/TRE-BCR-ABL* Mice

The transgenic EGFP^+^/*SCLtTA/TRE-BCR-ABL* mouse model was used to investigate the effect of LDE225 treatment on CML LSC *in vivo*. *Scl-tTa-BCR-ABL* mice in the FVB/N background were crossed with transgenic GFP-expressing mice (FVB.Cg-Tg [ACTB-EGFP] B5Nagy/J; The Jackson Laboratory)^31^. Bone marrow cells were obtained 4 weeks post induction, GFP^+^ cells were selected by flow cytometry and transplanted by tail vein injection (10^6^ cells/mouse) into wild-type FVB/N recipient mice, irradiated at 900 cGy, generating a large cohort of mice with similar time of onset of leukemia. Blood samples obtained 4 weeks post transplantation confirmed a neutrophilic leukocytosis in recipient mice. Mice were treated with nilotinib (50 mg/kg by gavage, daily), LDE225 (80 mg/kg by gavage, daily), LDE225 + nilotinib, or with vehicle alone (control). After 3 weeks of treatment, animals were euthanised and marrow content of femurs and tibiae, spleen cells and blood obtained. Total white cell count (WCC), GFP-expressing WCC, myeloid cells, and GFP^+^ progenitors and stem cells were measured by flow cytometry. Survival was assessed in a subset of mice for 120d post discontinuation of treatment. Spleen and BM cells from a subset of mice in each arm were pooled and 5 × 10^6^ cells/mouse (8 mice/condition) were transplanted into wild-type FVB/N recipient mice irradiated at 900 cGy. Engraftment was monitored by drawing peripheral blood (PB) every 4 weeks. The percentage of GFP^+^ cells in PB was analyzed by flow cytometry.

### Statistical analysis

Statistical analyses were performed using the Student’s T-test or ANOVA as necessary P ≤ 0.05 was considered statistically significant.

## Additional Information

**How to cite this article**: Irvine, D. A. *et al*. Deregulated hedgehog pathway signaling is inhibited by the smoothened antagonist LDE225 (Sonidegib) in chronic phase chronic myeloid leukaemia. *Sci. Rep*. **6**, 25476; doi: 10.1038/srep25476 (2016).

## Supplementary Material

Supplementary Information
