# Supplementary material for: Deregulated hedgehog pathway signaling is inhibited by the smoothened antagonist LDE225 (Sonidegib) in chronic phase chronic myeloid leukaemia
Source: Sci Rep. 2016 May 9;6:25476. doi: 10.1038/srep25476 (PMC4860619; doi:10.1038/srep25476)

**Supplementary Information**

**Deregulated hedgehog pathway signaling is inhibited by the smoothened antagonist LDE225 (Sonidegib) in chronic phase chronic myeloid leukemia**

David A. Irvine, Bin Zhang, Ross Kinstrie, Anuradha Tarafdar, Heather Morrison, Victoria L. Campbell, Hothri A. Moka, Yinwei Ho, Colin Nixon, Paul W. Manley, Helen Wheadon, John R. Goodlad, Tessa L. Holyoake, Ravi Bhatia, Mhairi Copland

**Inventory of Supplementary Information**

Supplementary Tables 1-5

Supplementary Figures 1-6

**Supplementary Table 1: Patient samples utilised for assessment of BCR-ABL by FISH in subpopulations.** Patient samples utilised for sub-population analysis (Supplementary Figure 1), percentage Ph^+^ by FISH in each sorted subpopulation and clinical outcome data.

| **Patient** | **FISH Positive (%)** | | | | **Clinical Outcome** |
| --- | --- | --- | --- | --- | --- |
|  | **HSC** | **CMP** | **GMP** | **MEP** |  |
| **CP CML 1** | **98** | **100** | **100** | **99** | **Alive and in CMR post IM followed by RISCT and DLI (clinical trial)** |
| **CP CML 2** | **89** | **90** | **89** | **90** | **Alive and in CMR post IM followed by RISCT and DLI (clinical trial)** |
| **CP CML 3** | **100** | **91** | **98** | **100** | **Alive and in MMR on IM** |
| **CP CML 4** | **98** | **97** | **100** | **100** | **Alive; IM resistance; in CCR on dasatinib** |
| **CP CML 5** | **87** | **94** | **91** | **97** | **Alive; CHR but not CCR; on IM 600mg** |

CMR; complete molecular response, IM; imatinib, RISCT; reduced intensity stem cell transplant, DLI; donor lymphocyte infusion, MMR; major molecular response, CCR; complete cytogenetic response, CHR; complete hematologic response.

**Supplementary Table 2: Details of antibodies used for immunocytochemistry of bone marrow sections.**

| **Antibody** | **Species** | **Dilution** | **Manufacturer**  **(Catalogue number)** |
| --- | --- | --- | --- |
| **α-tubulin** | **Mouse** | **1/500** | **Sigma T6793** |
| **γ-tubulin** | **Mouse** | **1/100** | **Sigma T5326** |
| **Alexa Fluor 568** | **Goat anti-Mouse** | **1/500** | **Invitrogen A21144** |
| **Alexa Fluor 488** | **Goat anti-Mouse** | **1/100** | **Invitrogen A21121** |

**Supplementary Table 3: De**tails of antibodies used for immunohistochemistry of bone marrow sections

| **Antibody** | **Control material** | **Dilution** | **Epitope retrieval**  **(Time in mins)** | **Manufacturer**  **(Catalogue number)** |
| --- | --- | --- | --- | --- |
| Anti-Sonic Hedgehog | Colon cancer | 1:100 | ER_1_ (20) | Millipore  (06-1106) |
| Anti-Indian Hedgehog | Colon cancer | 1:1000 | ER_2_ (20) | Millipore  (MABF23) |
| Anti-Desert Hedgehog | Testes | 1:1000 | EZ_1_ (10) | Millipore  (04-967) |

Both ER_1_ and ER_2_ solutions are specific for heat-induced epitope retrieval; ER_1_ is optimal at pH 6, ER_2_ optimal at pH 9. EZ: enzyme retrieval (Proteinase K).

**Supplementary Table 4: Human Taqman probes used.**

| Probe | Applied Biosystems identifier |
| --- | --- |
| *BCR-ABL* | ENP541F-MGB; 6FAM CCCTTCAGCGGCCAGT  ENF501: TCCGCTGACCATCAAYAAGGA  ENR561: CACTCAGACCCTGAGGCTCAA |
| *B2M* | Hs00984230_m1 |
| *CCNB1* | Hs01030099_m1 |
| *CCNB2* | Hs00270424_m1 |
| *CCND1* | Hs00765553_m1 |
| *DHH* | Hs00368306_m1 |
| *FOXM1* | Hs01073586_m1 |
| *GAPDH* | Hs02758991_g1 |
| *GLI1* | Hs01110766_m1 |
| *GLI2* | Hs00257977_m1 |
| *GLI3* | Hs00609233_m1 |
| *HHIP* | Hs01011015_m1 |
| *HRPT1* | Hs01003267_m1 |
| *IHH* | Hs01081801_m1 |
| *PTCH1* | Hs00970979_m1 |
| *PTCH2* | Hs00194904_m1 |
| *SHH* | Hs00179843_m1 |
| *SMO* | Hs01090242_m1 |
| *STIL* | Hs00161700_m1 |
| *SUFU* | Hs00171981_m1 |

**Supplementary Table 5: Murine Taqman probes used**

| Probe | Applied Biosystems identifier |
| --- | --- |
| *B2m* | Mm00437762_m1 |
| *CcnB1* | Mm00838401_g1 |
| *Cdc2* | Mm00772472_m1 |
| *Dhh* | Mm01310203_m1 |
| *Foxm1* | Mm00514924_m1 |
| *Gli1* | Mm00494645_m1 |
| *Gli2* | Mm01293111_m1 |
| *Gli3* | Mm00492345_m1 |
| *Ihh* | Mm00439613_m1 |
| *Ptch1* | Mm00436026_m1 |
| *Ptch2* | Mm00436047_m1 |
| *Shh* | Mm00436528_m1 |

**Supplementary Figure 1.** Quantitative expression of *BCR-ABL* in sorted subpopulations (HSC, CMP, GMP and MEP) from CD34^+^ cells from CP-CML patients at diagnosis (n=6).


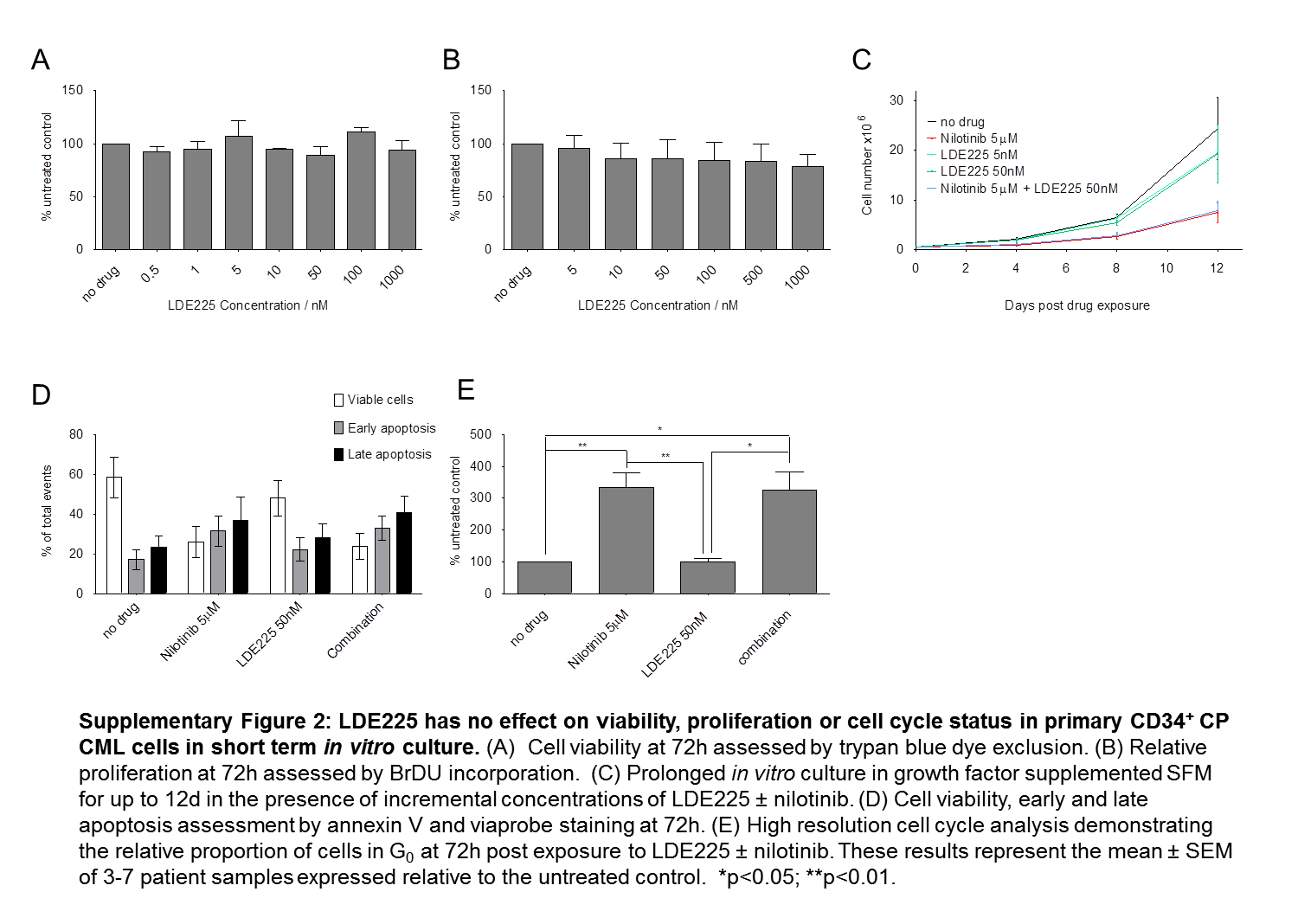


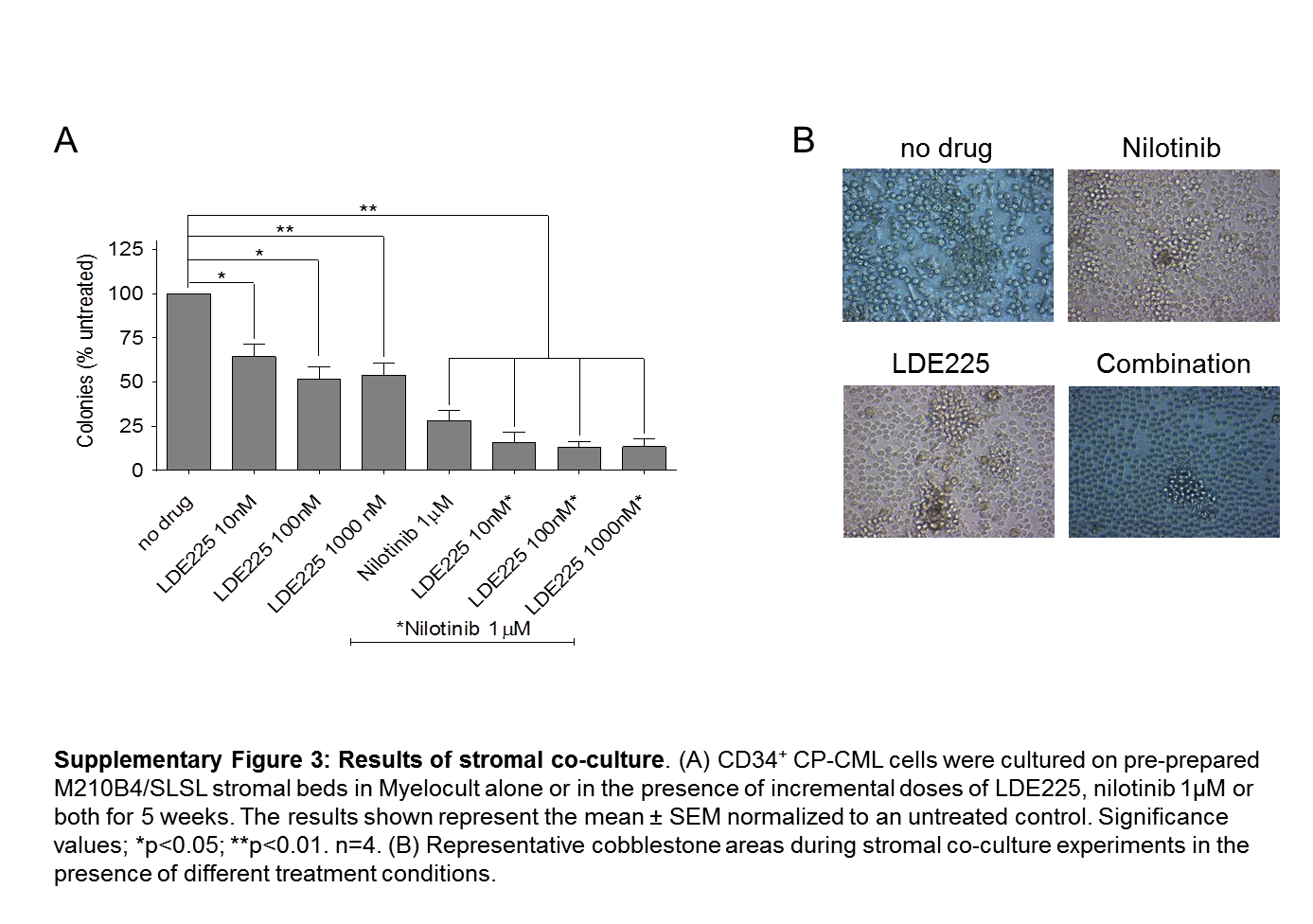


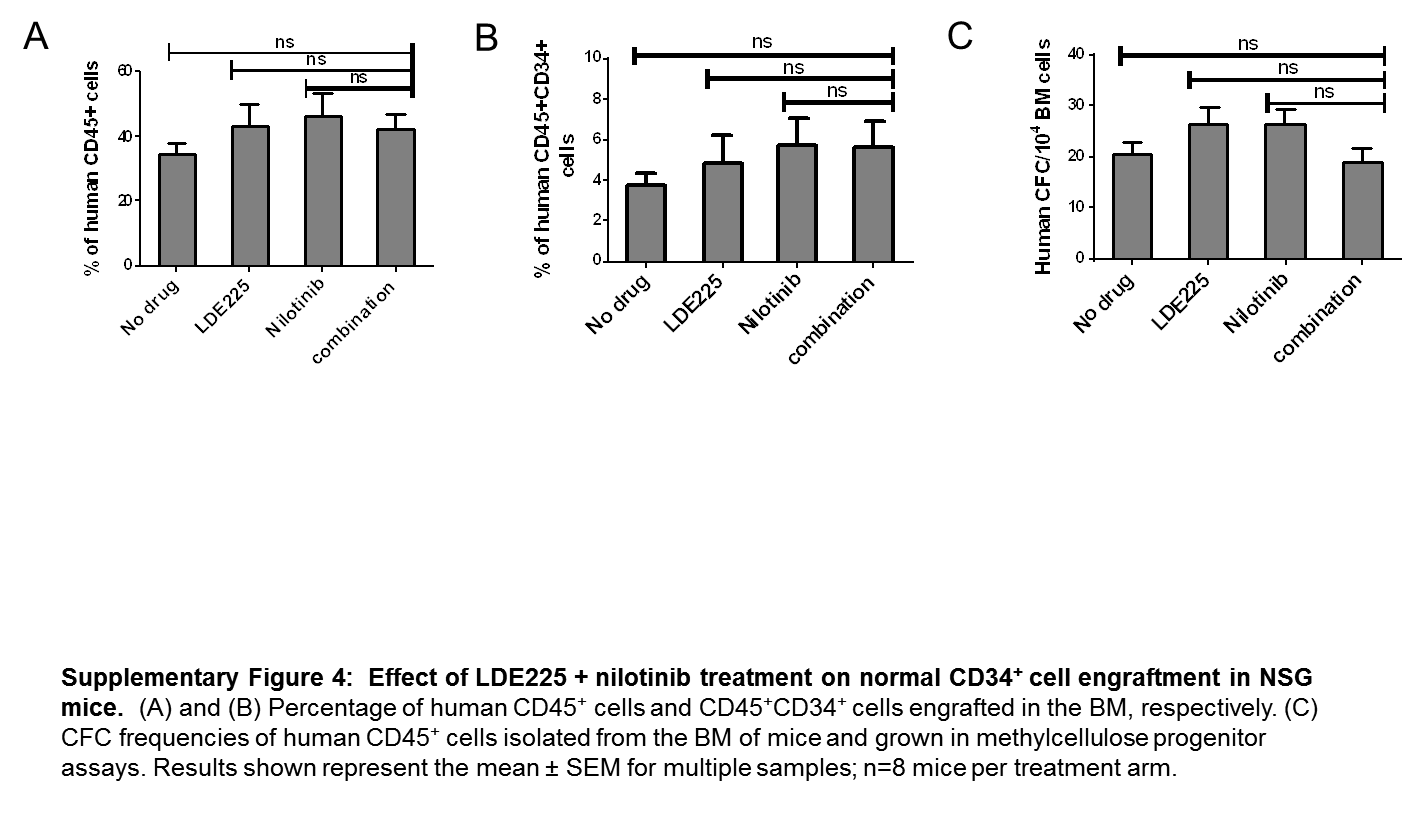


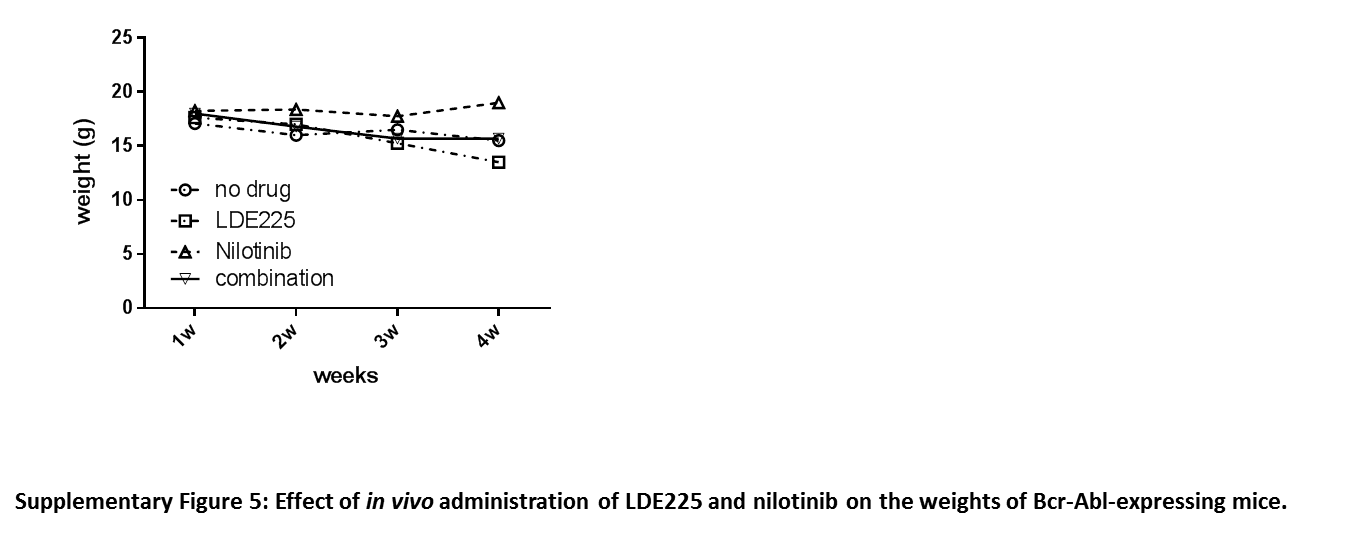


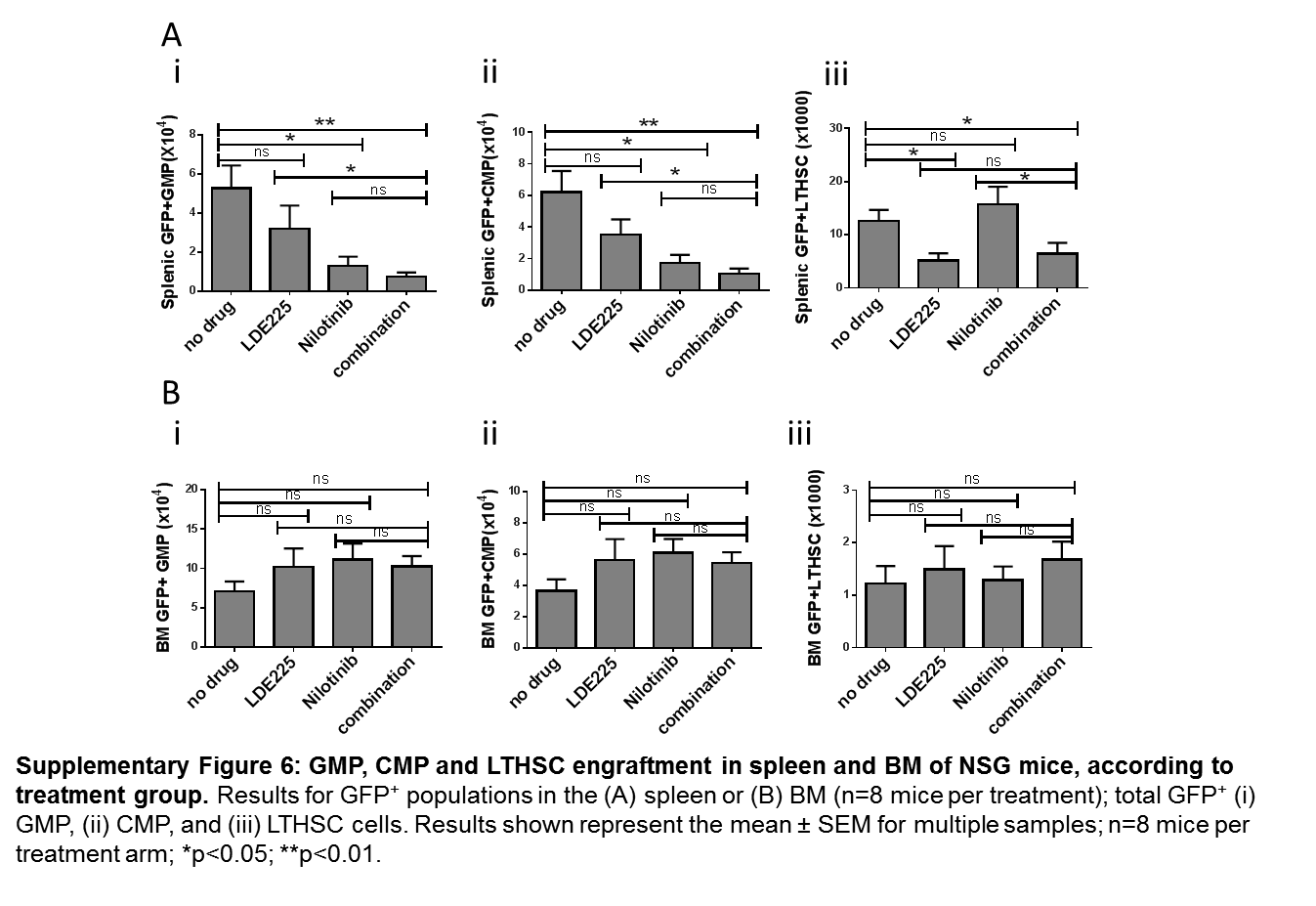

Supplement: Supplementary Information [file srep25476-s1.doc]
